# Reduced childhood social attention in autism model marmosets predicts impaired social skills and inflexible behavior in adulthood

**DOI:** 10.3389/fpsyt.2022.885433

**Published:** 2022-07-25

**Authors:** Akiko Nakagami, Miyuki Yasue, Keiko Nakagaki, Madoka Nakamura, Nobuyuki Kawai, Noritaka Ichinohe

**Affiliations:** ^1^Graduate School of Information Science, Nagoya University, Nagoya, Japan; ^2^Department of Ultrastructural Research, National Institute of Neuroscience, National Center of Neurology and Psychiatry, Kodaira, Japan; ^3^Department of Psychology, Japan Women’s University, Bunkyo-ku, Japan; ^4^Academy of Emerging Science, Chubu University, Kasugai, Japan

**Keywords:** early intervention, gaze direction, valproic acid, restricted behaviors, third-party reciprocity, inequity aversion, reversal learning, longitudinal study

## Abstract

Autism spectrum disorder (ASD) is a neurodevelopmental condition characterized by social and communication impairments and restricted and repetitive behavior. Although there is currently no established cure for ASD, early interventions for deficits of attention to other individuals are expected to reduce the progression of ASD symptoms in later life. To confirm this hypothesis and improve early therapeutic interventions, it is desirable to develop an animal model of ASD in which social attention is impaired in childhood and ASD-like social behavior is observed in adulthood. However, rodent models of ASD have difficulty in recapitulating the deficit of gaze-based social attention. In this study, we examined the direction of gaze toward other conspecifics during childhood and puberty in a three-chamber test setting using an ASD marmoset model produced by maternal exposure to valproic acid (VPA). We also conducted a reversal learning test in adult VPA-exposed marmosets as an indicator of perseveration, a core symptom of ASD that has not previously been investigated in this model. The results showed that time spent gazing at other conspecifics was reduced in VPA-exposed marmosets in childhood, and that mature animals persisted with previous strategies that required long days for acquisition to pass the test. In a longitudinal study using the same animals, deficits in social attention in childhood correlated well with ASD-like social disturbance (inequity aversion and third-party reciprocity) and inflexible behavior in adulthood. Since VPA-exposed marmosets exhibit these diverse ASD-like behaviors that are consistent from childhood to adulthood, VPA-exposed marmosets will provide a valuable means of elucidating mechanisms for early intervention and contribute to the development of early therapies.

## Introduction

Autism spectrum disorder (ASD) is a neurodevelopmental condition characterized by social and communication impairments and restricted or repetitive behaviors (1). The prevalence of ASD in children has been increasing over the years, and 1 in 44 children is currently diagnosed with ASD (2). However, at this time, there is no established cure for ASD. Research has shown that early interventions for ASD are more likely to have major long-term positive effects on symptoms and later skills than interventions at later ages (3, 4), and initiating such interventions is recommended as soon as a diagnosis of ASD is seriously considered or confirmed (4–7). A hypothesis for the mechanism of the early treatment effect is that the prodrome of ASD accelerates the development of later symptoms (8–11). In particular, early deficits in attention to the eyes and faces of others are thought to interfere with normal social input that promotes the proper development of social brain circuits (3, 11, 12). Furthermore, it is suspected that, after early brain plasticity is reduced, the abnormalities of aberrantly formed circuits will be difficult to reverse. To confirm these hypotheses and to establish effective therapeutic interventions, development of animal models of ASD with impaired social attention in childhood and higher-order ASD-like behavior in adulthood is desirable.

Rodent models of ASD have provided much insight into ASD to date. However, rodent models have difficulty in recapitulating the deficits in gaze-based social attention and impairments in higher-order social skills. In addition, neurodevelopmental events between birth and weaning in rodents occur between mid-gestation and birth in primates (13), making it harder to match the age of human childhood in rodents. The common marmoset (*Callithrix jacchus*), the New World monkey, is a promising new model for ASD because of its rich social skills, including eye contact, vocal communication, and evaluation of third-party reciprocity (14–17). The early social environment of marmosets influences later social skill acquisition. For example, the interaction of marmoset offspring with their parents influences multiple aspects of their social behavior development, including the acoustic properties of their calls, advanced communicative behaviors such as turn-taking, and social play (18–21). Furthermore, marmosets are easy to handle, mature rapidly, and have high reproductive capacity. These characteristics suggest that the marmoset can be used to study the treatment of ASD through early modulation of social attention. We established an ASD marmoset model by administering valproic acid (VPA) *in utero* (22–24). VPA modifies gene expression in the developing fetal brain by inhibiting histone deacetylase (HDAC). Marmosets exposed to VPA showed atypical calls in childhood (24). As adults, VPA-exposed offspring did not qualify for advanced social testing in previous studies (22, 23), as they were unable to recognize whether or not another individual reciprocated (22). Inequity aversion, which is hypothesized to have evolved in conjunction with cooperation (25), is not present in VPA-exposed marmosets (23) as the same in individuals with ASD (26, 27). Moreover, the transcriptome of the marmoset cortex model has been shown to reproduce the features of human ASD better than rodent models (24). Morphological analysis of VPA-exposed marmosets has revealed insufficient commissural fibers and altered immune cells in the brain, similar to human ASD (28, 29).

In this study, we performed a three-chamber social test on VPA-exposed marmosets from childhood through puberty to examine deficits in social attention. In addition to the chamber preference of the model animals, the direction of gaze to other conspecifics was assessed. We also conducted a reversal learning test in an adult marmoset model as an indicator of preservation, a core symptom of ASD that has not been previously investigated in this animal model. The results showed that VPA-exposed marmosets exhibited reduced social attention in childhood, and that mature animals persisted with previous strategies that required long days for acquisition to pass the test. In a longitudinal study using the same animals, social attention deficit in childhood correlated well with ASD-like social disturbance (inequity aversion and third-party reciprocity) (22, 23) and inflexible behavior in adulthood. Since VPA-exposed marmosets exhibit these diverse ASD-like behaviors that are consistent from childhood to adulthood, VPA-exposed marmosets will provide a valuable means of elucidating mechanisms for early intervention and contribute to the development of early therapies.

## Materials and methods

All experimental and animal care procedures were performed in accordance with NIH guidelines and the Guide for Care and Use of Laboratory Primates published by the National Institute of Neuroscience, National Center of Neurology and Psychiatry, and were approved by the Animal Research Committee at the National Institute of Neurosciences in Tokyo, Japan. Marmosets were bred in the National Center of Neurology and Psychiatry, kept under a 12 h/12 h light/dark cycle, and provided food (CMS-1, CLEA Japan) and water *ad libitum*. Temperature and humidity were maintained at 27–30°C and 40–50%, respectively. To produce marmosets that were prenatally exposed to VPA, serum progesterone levels in the female marmosets were monitored once a week to determine the timing of pregnancy. We took a minimum amount of blood (<0.1 mL) for the assay and provided the marmoset with extra nutrition after blood collection. In addition to the blood progesterone level, pregnancy was further confirmed by palpation and ultrasound monitoring (Ultrasound Scanning; Xario, Toshiba Medical Systems Corp., Tochigi, Japan). We administered 200 mg/kg of VPA (Sigma–Aldrich, St. Louis, MO, United States) seven times from day 60 to 66 after conception. The dose was calculated from the maximum dose used in human patients (1,200 mg/day) by considering the difference in body surface area between marmosets and humans (30). The time of VPA administration to be comparable to embryonic day 12 (E12) in rodent models, the period in which VPA induction produced the most prominent abnormality in social behavior without any physical deformity (31). The dose per body weight used in this marmoset experiment is considerably less than the dose used to create the autism model rodents. However, our previous study showed that the abnormalities in cortical gene expression in VPA-exposed marmosets with the doses used in this study are much closer to those in human ASD than in VPA-exposed ASD model rats (24). It has also been shown that the same dose of VPA can produce a model macaque monkey with autism-like behavior (32). To prevent vomiting, marmosets were first given bread, and VPA was delivered to the stomach 30 min later through a 4-Fr feeding tube (Atom Medical Corp, Tokyo, Japan) placed through the mouth, down the esophagus, and into the stomach. This procedure resulted in no obvious malformations or deformities. In this study, nine UE (unexposed) marmosets (three male and six females) and six VPA (prenatal exposure to VPA)-exposed marmosets (two male and four females) ranging from 15 weeks to 2.2 years of age were used. UE marmosets were born from four mothers and VPA-exposed marmosets were born from three mothers. There were no significant differences in weight between UE and VPA-exposed marmosets at 3 months old, 6 months old, and adult (mean ± SD: unit = g: 3 months old, UE, 182 ± 28, VPA, 160 ± 15; 6 months old, UE, 253 ± 34, VPA, 248 ± 33; adult, 359 ± 22, VPA, 404 ± 79) (see Supplementary Table for details).

### Three-chamber test

**FIGURE 1:**
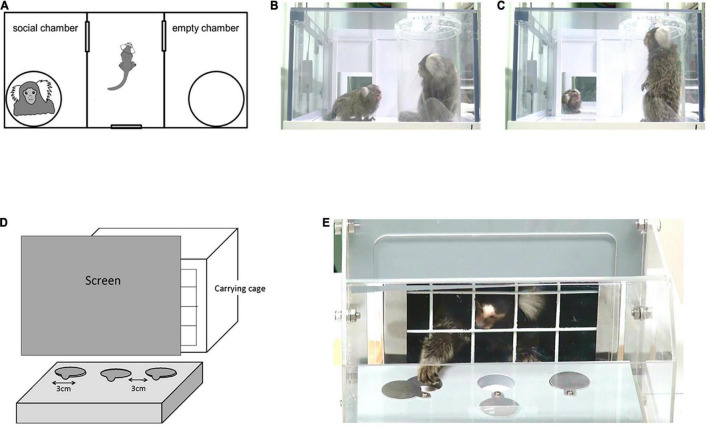
**(A)** Apparatus layout for the three-chamber test (88 cm W × 42 cm D × 22 cm H). Three interconnected chambers are separated by two manually operated sliding doors. An unfamiliar adult marmoset was placed in one of the side chambers, in a clear plastic tube with air holes on the lid. An empty tube was placed in the other side chamber. In the test phase, a subject marmoset entered the central chamber voluntarily and the sliding doors were closed. Both doors to the side chambers were then opened for the animal to explore the entire test apparatus. **(B,C)** Representative images of social gazing at each chamber. **(B)** The duration of social gazing in the social chamber was measured. **(C)** The duration of social gazing in the central chamber was measured. **(D)** Apparatus layout for the reversal learning test. The experiment was conducted in a Wisconsin General Test Apparatus (WGTA) for marmosets. The stimulus tray of the stimulus presentation chamber contained three wells in a row, each 3 cm in diameter. **(E)** Marmosets were accustomed by training prior to the test to open one of the sliding covers of the two wells and take the hidden bait.

Six VPA-exposed marmosets (one male and five females) and five UE marmosets (one male and four females) were subjected to a three-chamber test during childhood (15–19 weeks of age) and puberty (29–41 weeks). During each period, the experiments were conducted once a week, five times per individual. The apparatus for the three-chamber test comprised a rectangular three-chamber box (88 cm W × 42 cm D × 22 cm H). The central chamber (25 cm × 40 cm) and the two side chambers (30 cm × 40 cm) were separated by 6-mm-thick opaque Plexiglas walls with a central opening (12 cm × 12 cm) that allowed free access to each chamber (Figure 1A). Opaque sliding doors were placed at these openings. The central chamber had an entrance gate (16 cm × 16 cm) with a transparent door. Subjects entered their carrying cages voluntarily, and they were taken into the experimental room. Then, they spontaneously entered the central chamber of the experimental box through the entrance gate, and the opaque door between each chamber was closed. Thus, the experimenters had no contact with the subjects. While the subject stayed in the center chamber, two identical transparent cylinders (15 cm in diameter and 20 cm in height), large enough to contain a single adult marmoset, were placed vertically inside the apparatus, one in each side chamber. The cylinders had removable lids with ventilation holes, and gel absorption sheets were placed below and above the cylinders to prevent the lid from opening. Each cylinder was invisible from the other side chamber, but was visible from the central chamber. One empty cylinder was immediately replaced with a cylinder containing an unfamiliar marmoset, which had been prepared in a separate room. The subjects could not see the cylinder exchange. Both doors to the side chambers were then opened for the subject to explore the entire test apparatus for 10 min. After a 5 min acclimation in the center chamber, the door to both side chambers was opened.

Five male and five female adult marmosets served as unfamiliar conspecific animals and met with target animals of the same sex only once. These unfamiliar adults were not kin of the subjects, and had no previous interactions with them that might have provided visual, auditory, or olfactory cues.

The time each subject spent in each chamber (social chamber, central chamber, and empty chamber) and the duration of head orientation to the unfamiliar conspecific were measured (Figures 1B,C). The time spent orienting the head to an unfamiliar conspecific is an important measure of social interest, because social attention in primate species relies heavily on visual cues; the subject does not necessarily need to come close to the unfamiliar marmoset. In this study, we used the orientation of marmosets’ heads to infer their gaze toward other individuals. This is justified because marmosets have more restricted eye motility than Old World monkeys and humans, as reflected by their oculomotor range (marmosets, ∼10°; macaques, 40–50°; humans, ∼55°) (33–35). In addition, animals with smaller heads, such as marmosets, are assumed to use head rotation rather than eye rotation to determine gaze, because the exertion from head rotation is smaller than that for animals with larger heads (33). Note, however, that the usage of head direction as an indicator for gaze is still limited in that we did not take eye direction into account. Behaviors were recorded using three video cameras. The top video camera (Sony, Handycam), located 73 cm above the apparatus, recorded the entire apparatus. Video cameras on each side captured activity near the cylinder and in the open space of the central chamber at a distance of 40 cm from the side chamber. All recordings were synchronized and analyzed using the Observer XT 11 software (Noldus Information Technology, Wageningen, Netherlands).

The duration between the onset and offset of each behaviors was calculated by two observers. Reliability was analyzed using two methods. The Duration/Sequence method calculates the extent to which the scored duration of a given event is comparable between observations. The statistics showed that the percentage agreement between the observers was 92.17% and Cohen’s Kappa was 0.90. The Frequency/Sequence method calculates the extent to which the scored frequency of given events is comparable between observations; we programmed a tolerance window of 1 s. The statistics showed that the percentage agreement between observers was 84.58% and Cohen’s kappa was 0.80).

### Position reversal learning

Twelve juvenile (1.5- to 2.2-year-old) marmosets (two male and four female UE marmosets plus one male and five female VPA-exposed marmosets) were used in the reversal learning task. The experiment was conducted using a Wisconsin General Test Apparatus (WGTA) modified for marmosets. The WGTA was composed of a stimulus presentation chamber (20.5 cm × 25.0 cm × 20.0 cm) attached to a carrying box (Figures 1D,E). The stimulus tray of the stimulus presentation chamber (23.0 cm × 19.5 cm) contained three wells (3 cm in diameter) with slidable covers in a row.

Prior to the test, each marmoset underwent pre-training. First, they were trained to enter the carrying cage (20.0 cm × 25.0 cm × 18.5 cm) voluntarily. The monkeys learned to open the slidable covers on the two wells to pick up a hidden food reward (a piece of sponge cake; 0.5 cm × 0.5 cm × 0.5 cm). The test was initiated when the marmoset could reliably obtain a food reward from the left or right presentation well.

The test consisted of three phases: the first phase of discrimination learning (Phase 1), first reversal learning phase (Phase 2), and second reversal learning phase (Phase 3). The task involved positional learning, in which the marmoset had to learn to open either of the two positions (left or right) to be rewarded. One session of 20 trials per day was conducted. The interval between sessions was aimed at 2–3 days (mean ± SD: UE marmosets, 2.50 ± 0.20; VPA-exposed marmosets, 2.54 ± 0.14: no statistical difference: *p* = 0.68). The inter-trial interval was 5 s. When 90% accuracy was reached in three consecutive sessions in Phase 1, the correct position was reversed on the next experimental session (Phase 2). When the marmoset reached the same criterion, the reward condition was reversed again (Phase 3). If the marmoset did not displace the cover for 60 s or opened the incorrect position cover, the trial was recorded as an error.

### Correlation across varieties of behavior tasks in unexposed and valproic acid-exposed marmosets

The results of each behavioral test were scored as follows in order to examine correlations. (1) Social attention score were defined by the duration of attention toward the unfamiliar conspecific. (2) The inequity aversion score. Our previous study revealed that VPA-exposed marmosets failed to exhibit other-regarding behaviors (23). The unexposed marmosets refused to execute a simple task when they witnessed their partner receiving a more attractive reward for equal effort (inequity aversion). This inequity aversion was not observed in unexposed marmosets when the partner was absent. In contrast, VPA-exposed marmosets continuously executed the task, irrespective of their partners’ reward conditions. For this inequity aversion test, the ratio of food acceptance in the equity condition minus the ratio of food acceptance in the inequity condition was defined as the inequity aversion score. (3) The reciprocity evaluation score. The UE marmosets responded negatively to non-reciprocal human actors when they observed an unfair exchange between third parties who had no direct relevance to the marmosets, whereas VPA-exposed marmosets did not exhibit any differential behavior between non-reciprocal and reciprocal conditions. For this third-party reciprocity test, the ratio of food acceptance from a reciprocator in the reciprocal condition to the ratio of food acceptance from a non-reciprocator in the non-reciprocal condition was defined as the reciprocity evaluation score.

### Statistical analysis

All statistical analyses were performed using JMP 16 software (SAS Institute, Cary, NC, United States). All values are expressed as the mean ± standard deviation, and *p*-values less than 0.05 were considered statistically significant. A non-parametric analysis of variance by a downloadable program (ARTool) (36) was performed using R if the data were not normally distributed by the Shapiro–Wilk test, or if unequal variances were found by the *F*-test.

## Results

### Three-chamber test

**FIGURE 2:**
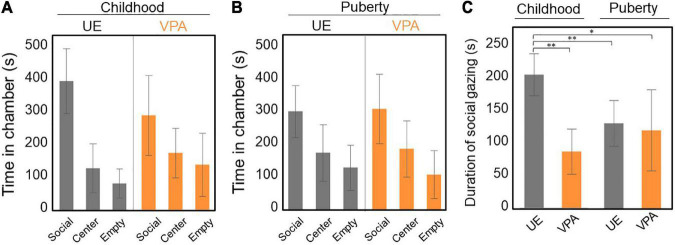
**(A–C)** Three-chamber test conducted both in childhood (15–19 weeks of age) and puberty (29–41 weeks of age). **(A)** Time spent in each chamber. The unexposed (UE) group spent a longer time in the social chamber than the valproic acid (VPA)-exposed group, but the difference is not significant. Two-way repeated-measures ANOVA, *n* = 5 for UE, *n* = 6 for VPA exposed. **(B)** Time spent in each chamber. There was no significant difference between the three chambers. Two-way repeated-measures ANOVA, *n* = 5 for UE, *n* = 6 for VPA exposed. **(C)** The duration of social gazing in childhood in the UE group is significantly longer than that in the other three groups. Two-way repeated-measures ANOVA [*F*(1,9) = 21.5525, *p* = 0.0012] followed by Tukey’s HSD *post hoc* test, *n* = 5 for UE, *n* = 6 for VPA exposed. *p < 0.05 and **p < 0.005. Error bars represent SD.

Marmosets underwent the three-chamber test five times between 15 and 19 weeks of age (childhood). Both VPA-exposed and age-matched UE marmosets spent more time in the social chamber with an unfamiliar marmoset than in the other two chambers. However, there was no statistical difference between model and control marmosets in terms of the time spent in each chamber (*p* = 0.185, two-way repeated-measures ANOVA, Figure 2A). Marmosets in puberty underwent the three-chamber test once a week for five consecutive weeks between 29 and 41 weeks of age. As with the childhood results, there was no significant difference between the time spent in each chamber (*p* = 0.921, two-way repeated-measures ANOVA, Figure 2B).

We also examined the duration of social gazing in marmosets in childhood and puberty. Two-factor repeated measures ANOVA showed a significant difference in the group factor [*F*(1,9) = 7.3216, *p* = 0.0242] and the interaction effects [*F*(1,9) = 21.5525, *p* = 0.0012]. We did not find any significant difference in the age factor [*F*(1,9) = 3.1127, *p* = 0.1115]. Multiple comparisons by Tukey’s *post hoc* test revealed that the duration of social gazing was significantly longer in UE marmosets in childhood than in both VPA-exposed marmosets in childhood and in UE marmosets in childhood and puberty (Figure 2C).

### Position reversal learning

In this study, we sought to assess the long-term cognitive inflexibility associated with obsession with habit and sameness seen in individuals with ASD in VPA-exposed marmosets (Figure 1B). Thus, we employed reversal learning, in which the reward side is reversed during the session following the session in which the criterion is met. Sessions were scheduled about 2 days apart. This differs from a typical short-term cognitive flexibility test, in which food positions are reversed multiple times during the day (37–39).

**FIGURE 3:**
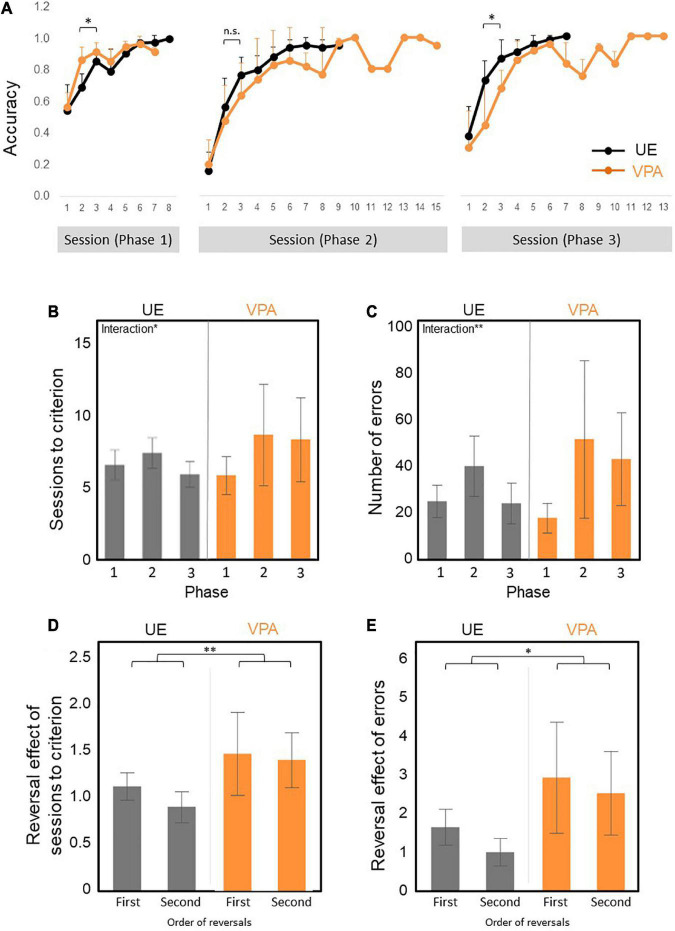
**(A–E)** Reversal learning results across three phases. Phase 1 is acquisition trials. Phase 2 is reversal learning. Phase 3 is re-reversal learning. *N* = 6 for UE, *n* = 6 for VPA exposed. **(A)** The curve of the percentage of correct answers across all trial phases. VPA-exposed marmosets performed better than UE marmosets for the first 2–3 days of Phase 1, but worse in Phase 2 and Phase 3. However, significant differences are observed only in Phase 1 and Phase 3. There are statistically significant phase effect and interaction **(B)** Numbers of sessions required to achieve the test criteria. There are statistically significant phase and interaction effect. **(C)** Number of errors required for attainment criteria. There are statistically significant phase and interaction effect. **(D)** First and second reversal effects of the number of sessions required to achieve the test criteria. There is a statistically significant group effect. **(E)** First and second reversal effects of the number of errors to reach the test criteria. There are statistically significant group and phase effect. *p < 0.05 and ^**^p < 0.01. Error bars represent SD.

First, we examined the percentage of correct responses of the first 2–3 sessions in the initial discrimination phase (Phase 1), the reversal phase (Phase 2), and the re-reversal phase (Phase 3; Figure 3A) as an index for pace of learning. The percentage of correct response of the first 2–3 sessions of the Phase1 was statistically significantly higher in VPA-exposed marmosets than the UE marmoset [group factor *F*(1,10) = 8.0328, *p* = 0.0177] and the interaction effects [*F*(1,10) = 8.4483, *p* = 0.0157: two-factor repeated measures ANOVA], and that of the Phase 3 was statistically significantly lower [group factor *F*(1,10) = 7.37972, *p* = 0.0217] and the interaction effects [*F*(1,10) = 12.43674, *p* = 0.0054: non-parametric factorial ANOVA]. The percentage of correct response of the first 2–3 sessions of the Phase 2 tended to be smaller in the VPA-exposed marmoset, although there was no statistical significance [group factor *F*(1,10) = 1.0478, *p* = 0.3301) and the interaction effects [*F*(1,10) = 0.8420, *p* = 0.3804: two-factor repeated measures ANOVA]. These results indicate that the VPA-exposed marmoset learned new skills more quickly but tended to adhere to previous learning.

Second, we have examined the number of sessions and the number of errors required to reach the criteria as an index of learning efficiency. A non-parametric factorial ANOVA was performed to analyze the effect of VPA exposure group and learning phase on the number of necessary sessions. The result showed a significant difference in the phase factor [*F*(2,20) = 4.64403, *p* = 0.022] and the interaction effects [*F*(2,20) = 4.59538, *p* = 0.0228]. We found no significant differences in the group factor [*F*(1,10) = 0.18683, *p* = 0.6747] (Figure 3B). The numbers of errors required to reach the criteria produced the similar patterns (Figure 3C). A non-parametric factorial ANOVA showed a significant difference in the phase factor [*F*(2,20) = 24.57343, *p* = 0.000008] and the interaction effects [*F*(2,20) = 3.1862, *p* = 0.0629]. There were no significant differences in the group factor [*F*(1,10) = 0.76751, *p* = 0.4015] (Figure 3C). The significant main effect of phase on the learning efficiency was likely due to the tendency for the learning efficiency to be lower in phase 2 than in phase 1 for both groups (Figures 3B,C), while the significant interaction effect on learning efficiency may be explained by the tendency for learning efficiency to be higher in Phase 1 and lower in Phase 3 in VPA-exposed marmosets (Figures 3B,C).

Third, we defined the first and second reversal effects as the number of sessions needed to reach the criteria in Phases 2 and 3 minus the number of sessions needed to reach the criteria in Phase 1 divided by the number of sessions reached in Phase 1, respectively (Figures 3D,E). The first and second reversal effects of the number of sessions required to reach the criteria were larger in VPA-exposed marmosets than UE marmosets [group factor *F*(1,10) = 12.2932, *p* = 0.0057: A non-parametric factorial ANOVA] (Figure 3D). In addition, the first and second reversal effects of the number of errors required to reach the criteria were also larger in VPA-exposed marmosets than UE marmosets [group factor *F*(1,10) = 8.7874, *p* = 0.014] and phase factor [*F*(1,10) = 8.9203, *p* = 0.014: A non-parametric factorial ANOVA]. These results suggested that VPA-exposed marmosets exhibited a decrease in performance on the test after reverse, and further endorsed the behavior perseverance of the VPA-exposed marmosets.

### Correlations across varieties of behavioral tests in unexposed and valproic acid-exposed marmosets

**FIGURE 4:**
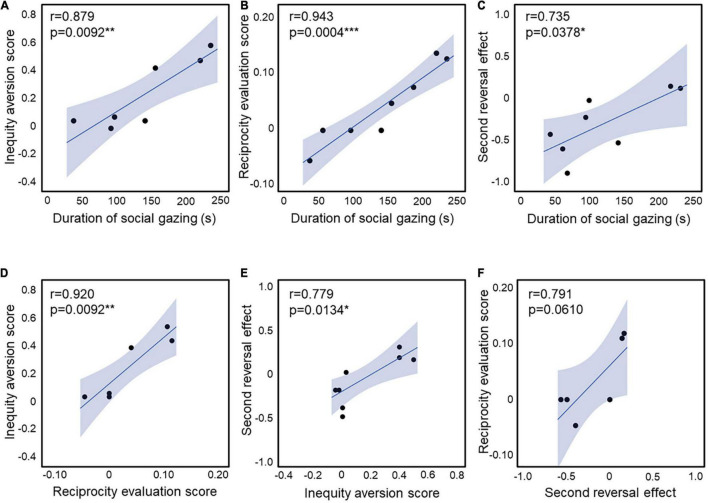
**(A)** Correlation between the inequity aversion score and the duration of social gazing in childhood (*n* = 3 for UE, *n* = 4 for VPA exposed). **(B)** Correlation between the reciprocity evaluation score and the duration of social gazing in childhood (*n* = 4 for UE, *n* = 4 for VPA exposed). **(C)** Correlation between the second reversal effect and the duration of social gazing in childhood (*n* = 2 for UE, *n* = 6 for VPA exposed). **(D)** Correlation between the inequity aversion score and the reciprocity evaluation score (*n* = 3 for UE, *n* = 3 for VPA exposed). **(E)** Correlation between the second reversal effect and the inequity aversion score (*n* = 5 for UE, *n* = 4 for VPA exposed). **(F)** Correlation between the reciprocity evaluation score and the second reversal effect (*n* = 2 for UE, *n* = 4 for VPA exposed). *p < 0.05, **p < 0.01, and ***p < 0.001. Error bars represent SD.

The social attention score in UE and VPA-exposed marmosets during childhood in the three-chamber task correlated with the behavioral scores of inequity aversion (23) in adults (*r* = 0.879, *p* = 0.0092; Figure 4A). These short attention scores were correlated with the discrimination scores of the third-party evaluation test (22) in adults (*r* = 0.943, *p* = 0.0004; Figure 4B). The second reversal effect in adults was correlated with the social attention score during childhood (*r* = 0.879, *p* = 0.0092; Figure 4C). There was also a strong correlation between inequity avoidance and third-party reciprocity assessment scores (*r* = 0.920, *p* = 0.0092; Figure 4D). The strong correlations between the scores of the three social behavioral tests (social attention during childhood, inequity avoidance, and third-party reciprocity in adulthood) confirm the robust social impairment of VPA-exposed marmosets. In addition, the second reversal effect score was correlated with inequity aversion (*r* = 0.779, *p* = 0.0134; Figure 4E). There was a non-significant correlation between the reciprocity evaluation score and the second reversal effect (*r* = 0.791, *p* = 0.0610; Figure 4F). The social attention score during puberty was not correlated with any of the other behavioral tests (Table 1).

**TABLE 1:** Pearson correlation coefficient *r* between the durations of social gazing in childhood and puberty, the reciprocity evaluation score, the inequity aversion score, and the second reversal effect of the reversal learning test.

	Duration of social gazing-childhood-	Duration of social gazing-puberty-	Reciprocity evaluation score	Inequity aversion score	Second reversal effect
Duration of social gazing-childhood-		0.413	0.943	0.879	0.735
Duration of social gazing-puberty-			0.319	–0.047	0.284
Reciprocity evaluation score	***			0.920	0.791
Inequity aversion score	**		**		
Second reversal effect	*			*	0.779

P-values indicate statistical significance *p < 0.05, **p < 0.01, ***p < 0.001.

## Discussion

This study showed that during childhood, VPA-exposed marmosets spent less time gazing at other individuals than UE marmosets. This short social attention time in childhood correlated well with the impairment of two higher-order social abilities (inequity aversion and third-party reciprocity) in VPA-exposed marmosets during adulthood (22, 23). These results are consistent with the notion that early deficits in social gazing are associated with worse ASD symptoms later in life (11, 12, 40). The VPA-exposed marmoset in adulthood have difficulty shifting from the effects of past long-term learning, and this deficit was also associated with a shorter duration of gaze toward other individuals in childhood.

The disruption of gaze toward other conspecifics in VPA-exposed marmosets during childhood is consistent with studies of human children with ASD. Analyses of home movies showed that infants up to 6 months of age (9) and from 12 to 30 months of age (8) who were diagnosed with ASD spent less time looking at and orienting toward people than normal children. Also, many previous studies using eye-tracking systems found that children and adults with ASD spent less time looking into the eyes of others (10, 12, 41). However, in this study, there was no difference in social attention between UE and VPA-exposed marmosets in puberty. This may be because social gazing in UE marmosets becomes shorter in puberty than in childhood (Figure 2C), making the differences between the groups too small to detect. Adult marmosets share food items with immature marmosets (i.e., those in childhood; 10–18 weeks of age), but they are only one-seventh as likely to share food with animals that have passed puberty (24 weeks of age) (42). Reduced reward values of adult marmosets for pubertal marmosets may be associated with reduced gaze duration. As a next step, it would be worthwhile to perform a long-term investigation of the degree of attention paid by UE and VPA-exposed marmosets to the eyes of other animals using more sophisticated system, such as an eye tracking system. An eye-tracking system revealed social target avoidance in adult ASD macaques models induced by immune activation or exposure to VPA *in utero* (32, 43), and showed social feature preference in subadult macaques in an MeCP2 mutant ASD model (44). However, the gaze of these ASD model monkeys has not yet been examined in childhood. There are no studies in which gaze has been used to evaluate social attention in rodent models.

In young children (12–24 months) with ASD, abnormalities in eye contact have been correlated with deficits in both empathy and prosocial behavior in preschool (45). Longitudinal studies have shown that joint attention is associated with social cognitive outcomes such as language development (46) and theory of mind (47). Early delays in basic face processing, including attention to faces, have been considered to contribute to an atypical trajectory of social communication skills in individuals with ASD (11). In the marmosets examined in this study, weak attention to others in early childhood was correlated with performance in two social cognitive tasks in adulthood. This provides evidence that the behavioral phenotype in this animal model is robust as a social disorder and is consistent with the notion that weak attention to others in early childhood amplifies the trajectory of autistic symptom development later in life (11, 12, 40, 48).

The VPA-exposed marmosets showed a quicker increase in performance during the first discrimination phase (Phase 1). Consistent with this finding, strong memory is often seen in children with ASD (49). For example, superior performance for individuals with high functioning ASD was frequently reported in tasks involving recall of a path in maze, and shorter learning times in a map learning task (50). Individuals with ASD can also remember the exact location of objects in places they have only been once. Furthermore, the higher capability of VPA-exposed marmosets in Phase 1 suggested that their previously reported failure in social tasks (22, 23) cannot be ascribed to a decline in intellectual ability.

The VPA-exposed marmosets exhibited perseveration, as measured by the first and second reversal effects. They had a greater reduction in learning efficiency after reversal relative to learning efficiency before reversal. A slower pace of learning in Phase 3 also supported response rigidity in these animals. Another possible interpretation of the reversal learning phenotype is that VPA-exposed marmosets had an impairment in consolidating new information. In fact, it has been reported that individuals with ASD have a learning pace similar to that of control subjects after reversal, but that there was a deficit in the maintenance of acquired learning (51). However, it should be noted that in that report of human ASD, reversal occurred several times per day, whereas in our task reversal occurred only once a month at most. Consistent with the phenotype of VPA-exposed marmosets, individuals with ASD have persistent habits and a desire for sameness, and resist changes in the environment and their behaviors (52). In addition, a previous study revealed that during childhood, VPA-exposed marmosets had a call pattern with low entropy that was biased toward one type of call (phee call) (24). This is consistent with the frequent involuntary repetition of words and phrases (palilalia) in people with ASD (53). These results suggest that in both childhood and adulthood, VPA-exposed marmosets are primed for restricted and repeated behavior, one of the two core symptoms of ASD.

In VPA-exposed marmosets examined here, gazing time scores in childhood and social skill scores in adulthood correlated with adult rigidity scores, suggesting that their behavioral phenotypes may be rooted in the same brain pathophysiology. The medial prefrontal cortex has been implicated in sociality and cognitive flexibility (50, 53, 54). Serotonin depletion in the prefrontal cortex severely impairs reversal learning performance in marmosets (37). Interestingly, downregulation of the serotonin receptor–encoding gene *HTR5A* has been reported in the cortex of VPA-induced marmosets (24).

## Conclusion

The results of this study suggest that VPA-exposed marmosets will provide a useful platform for investigating how early rectification of weak social attention impacts the progression of ASD symptoms, and will help in the development of effective early treatments. Previous synaptic and gene expression study in VPA-exposed marmosets has indicated that the brains of human children with ASD may exhibit aberrant plasticity during a critical period (24). Atypical plasticity itself may alter the trajectory of the normal development of social brain circuits, even if social experiences are normalized by behavioral interventions. Drugs that correct anomalous circuit plasticity combined with behavioral interventions may further enhance the effectiveness of early treatment.

### Limitation

In this study, due to the availability of animals, the subject animals were predominantly female, both UE and VPA-exposed marmosets. In humans, ASD is more frequent in males, with an approximate sex ratio of 3 to 1. However, our previous studies in VPA-exposed marmosets have shown no gender differences in gene expression, cell biology, or brain morphology. However, it is possible that a stronger phenotype could be detected by increasing the male population. In addition, we could not evaluate the gaze direction of the marmosets in the cylinders because their heads were often not visible, even from the three video cameras. Eye contact, or joint attention, is the exchange of gaze between two individuals, and its impairment is a major symptom of ASD. As marmosets are one of very few non-human primate species that engage in eye contact (16), it would be valuable to monitor the gaze of both target animals and partnered unfamiliar conspecifics in the future. Furthermore, to reduce stress in young marmosets, we did not conduct a control experiment to confirm that impaired head orientation to the conspecifics of VPA-exposed marmosets reflected an attention disorder rather than a physical defect (e.g., poor eyesight). For example, instead of an adult marmoset, an experiment in which an object was placed in the cylinder was considered. However, the strong correlation between scores for head orientation to other individuals in childhood and scores for social experiments in adulthood supports the existence of impaired social attention in young VPA-exposed marmosets.

## Data availability statement

The raw data supporting the conclusions of this article will be made available by the authors, without undue reservation.

## Ethics statement

The animal study was reviewed and approved by the Animal Research Committee at the National Institute of Neurosciences in Tokyo, Japan.

## Author contributions

NK and NI designed the study. AN and MY performed the behavioral experiments. AN, MY, and MN analyzed the data. KN managed the production and physical condition of the animals. NK, NI, AN, MY, and MN wrote the manuscript. All authors have read and approved the final manuscript.
